# Supplementary material for: Control of anterior GRadient 2 (AGR2) dimerization links endoplasmic reticulum proteostasis to inflammation
Source: EMBO Mol Med. 2019 Apr 30;11(6):e10120. doi: 10.15252/emmm.201810120 (PMC6554669; doi:10.15252/emmm.201810120)
Supplement: Supplementary file 1 — Appendix [file EMMM-11-e10120-s001.pdf]

Maurel et al. – Appendix

Table of Content

Appendix Table S1: Candidate AGR2 dimer regulators …………………………………….. page 2
Appendix Table S2: Mpd1 ER-MYTHS interactions …………...………………………….. page 4
Appendix Table S3: Patient information ………………………..…...…………………………. page 5
Appendix Table S4: Primers used in this study …..………………....…………………………. page 6
Appendix Figures Legends ……………………….………………………………………… page 9
Appendix Figure S1……………………….……………………………….………. page 13
Appendix Figure S2……………………….……………………………….………. page 14
Appendix Figure S3……………………….……………………………….………. page 15
Appendix Figure S4……………………….……………………………….………. page 16
Appendix Figure S5……………………….……………………………….………. page 17
Appendix Figure S6……………………….……………………………….………. page 18
Appendix Reference ………………...………………….……………………………………. page 19

Appendix Table S1 - Candidate AGR2 dimer regulators. Candidate AGR2 homodimer enhancers (negative fold change) and inhibitors (positive fold change), ranked according to their statistical significance. Fold change calculation is based on the log2 transformed values, normalized according to the Quantile method and depicts the difference in Luciferase units between ERMIT and the counter-screen assay. The presented p-value is the optimal one among the three different normalization methods.

Symbol	Fold Change	CV based p-value
CD74	-0.58	0.000000004
UGGT1	-2.23	0.000000036
SRPRB	0.79	0.000002601
DMPK	0.90	0.000003670
RPN1	-1.05	0.000009407
CHERP	1.27	0.000023399
KTN1	-1.32	0.000061785
TMED2	-0.48	0.000101576
GRIN3A	0.87	0.000166860
ELOVL6	1.22	0.000216427
GANAB	-0.30	0.000319945
HERPUD1	-0.61	0.000420459
ANXA6	0.74	0.000636071
ADCY10	-1.17	0.000780833
RYR2	-0.21	0.001178128
SHISA5	-0.45	0.001333969
SYP	0.63	0.001577195
SLN	-0.91	0.005462889
P4HTM	0.89	0.005775801
PCYT1B	-0.78	0.007075369
SEC63	0.72	0.007623527
ACSL1	0.74	0.007861788
CDS1	-0.64	0.008581381
DNAJC10	-0.54	0.009679147
RTN2	-0.91	0.013635551
PEMT	-0.56	0.014933474
H6PD	-0.51	0.015130531
PLPP3	0.92	0.018123538
MAL	0.85	0.018499011
LMAN1	-0.79	0.022088191
RTN4	-0.84	0.024045311
CES3	0.60	0.026533589
ARL6IP1	-1.07	0.028561246
EIF3I	0.61	0.029330841
CH25H	0.45	0.032233404
SYVN1	1.07	0.032579678
ATP2A3	-0.67	0.032683782
EXTL2	-0.63	0.032886150
UBE2J1	-0.33	0.034806224
PSMG4	-0.48	0.039425145
ATP2A2	-0.54	0.040757411
MPDU1	-0.55	0.043304942
RTN1	-0.44	0.043780729
VPREB3	-0.38	0.043781190
PRNP	-0.58	0.044471339

**BCL2**	0.68	0.048881117
**TOR2A**	-0.31	0.048908822
**KDELC1**	-0.82	0.051698615
**SPCS1**	0.59	0.053850994
**AMFR**	-0.42	0.056806929
**SSR3**	-0.38	0.057859908
**AKAP6**	-0.55	0.060150351
**CKAP4**	0.92	0.062765024
**SCAP**	0.81	0.062942949
**ELOVL4**	-0.59	0.064278848
**EXTL3**	0.54	0.067157432
**HYOU1**	0.64	0.070778266
**SQLE**	0.54	0.070871370
**ERO1A**	0.70	0.071287505
**RRAS**	-1.17	0.071950710
**P4HA1**	0.26	0.076943536
**ERP44**	0.24	0.078356611
**ALG5**	-0.57	0.079767352
**HSP90B1**	0.67	0.081415226
**ERO1B**	-0.45	0.082442670
**BCAP31**	0.79	0.085393017
**CHMP5**	-0.50	0.088916903
**TXNDC12**	-0.94	0.091395094
**CACNA1S**	-0.56	0.096276611
**PCSK5**	0.45	0.098404888
**GAS1**	-0.09	0.098918875

Appendix Table S2 - Mpd1 ER-MYTHS interactions. Full length coding sequences were used except for Erp1 (luminal aa 1-188), Erp5 (luminal aa 1-180), and Erv25 (luminal aa 1-180).

Protein 1	#	Protein 2	#	Interaction	Evidence
**Mpd1**	Q12404	**Mpd1**	Q12404	Physical	This study
**Mpd1**	Q12404	**Erp1**	Q05359	Physical	This study
**Mpd1**	Q12404	**Erp5**	P38819	Physical	This study
**Mpd1**	Q12404	**Erv25**	P54837	Physical	This study
**Mpd1**	Q12404	**Gpi10**	P30777	Genetic	(Jonikas, Collins et al., 2009)
**Mpd1**	Q12404	**Jem1**	P40358	Genetic	(Costanzo, VanderSluis et al., 2016)

Appendix Table S3 - Patient information. Clinical information about Crohn's disease (CD), Ulcerative colitis (UC) and control patients analyzed in this study. *Montréal classification (Silverberg et al. Can J Gastroenterol. 2005 Sep;19 Suppl A:5A-36A) : For Crohn's disease location L1 = ileal, L2= colonic, L3 = ileocolonic, L4= upper-GI disease. For Ulcerative Colitis extension: E1 = proctitis, E2 = left sided colitis, E3 = pancolitis. ** All had normal colonic mucosa. Indications of colonoscopy : 19 adenoma screening, 5 rectal bleeding and 8 Irritable Bowel Syndrom.

	Crohn's Disease (n=40)	Ulcerative Colitis (n=8)	Controls** (n=32)
Males (n, %)	22 (55)	5	17 (53)
Age (median, ranges)	40 [18-67]	60 [36-74]	57 [27-87]
Location (Montreal classif*)	9 L1/20 L2/11 L3/0 L4	0 E1/ 3 E2/ 5 E3	NA
Active disease (n, %)	19 (47)	4	NA
Previous surgery (n, %)	11 (27)	2	NA
Active smoking (n, %)	14 (35)	0	missing data
Anti-TNF (n, %)	17 (42)	1	0
Immunosuppressant (n, %)	8 (20)	3	0
5-ASA (n, %)	8 (20)	4	0
Steroids (n, %)	2 (5)	0	0

Appendix Table S4 – Primers used in this study.

Specification	Sequence
hAgr2 E60A mutagenesis	(Forward 5'-3')- CTGGACTCAGACATATGAAGCAGCTCTATATAAATCCAAGAC
hAgr2 E60A mutagenesis	(Reverse 5'-3')- GTCTTGGATTTATATAGAGCTGCTTCTTCATATGTGTCCAG
AGR2 mutagenesis AXXS	(Forward 5'-3')- CTTGGATGAGGCCCCACACAG
AGR2 mutagenesis AXXS	(Reverse 5'-3')- CTGTGTGGGGCCTCATCCAAG
CD59 mutagenesis IRE1 cleavage site	(Forward 5'-3')- CAGGTCATAGCATTAAGTGCTACAAC
CD59 mutagenesis IRE1 cleavage site	(Reverse 5'-3')- GTTGTAGCACTTAATGCTATGACCTG
PCR mutagenesis IRE1 K599A	(Forward 5'-3')- AACCGCGACGTGGCCGTGGCGAGGATCCTCCCCGAGTGT
PCR mutagenesis IRE1 K599A	(Reverse 5'-3')- ACACTCGGGGAGGATCCTCGCCACGGCCACGTCGCGGTT
PCR mutagenesisIRE1 K599A	(Forward 5'-3')- GTGGCCGTGGCGAGGATCCT
PCR mutagenesis IRE1 K599A	(Reverse 5'-3')- GAGGATCCTCGCCACGGCCAC
ERN1 (from AA444) for AGR2 fusion in pcDNA5	(Forward 5'-3')- GGGGGTACCGGATCCATGGCTACCATCATCCTGAG
ERN1 (from AA444) for AGR2 fusion in pcDNA5	(Reverse 5'-3')- GGGCTCGAGCAGGGCGTCTGGAGTCACTGG
Scotin/ SHISA5	(Forward 5'-3')- GTGCATGCCCCTTATCCTCA<br>(Reverse 5'-3')- GGTAAGGTGGTGGGTACTGC
KTN1	(Forward 5'-3')- GCAAAACACAGCTGTTACAGGA<br>(Reverse 5'-3')- TGAGGGGGAAAAGAAGATGCC
PLP2	(Forward 5'-3')- AGGGGTACTGGCCTAATCG<br>(Reverse 5'-3')- CGAACGGGGAAGGTGACATA
CD74	(Forward 5'-3')- ATGCACAGGAGGAGAAGCAG<br>(Reverse 5'-3')- GGGCAGTTGCTCATTGTTGG
HYOU1	(Forward 5'-3')- TAGAGGAGCGCAAGAAGTGG<br>(Reverse 5'-3')- CCCTTGAGGAACATGCTGGA
ALG5	(Forward 5'-3')- TGGCTATAGCATGTGGATCTCG<br>(Reverse 5'-3')- AGGAACCACACCAGAAAGTGG
PSMG4	(Forward 5'-3')- CGACTCCATCCCCGTGTCTA<br>(Reverse 5'-3')- CCAGTAGAGGTCGTGTCGGA
TXNDC12	(Forward 5'-3')- TGGAAACCCCAGCTACAAGT<br>(Reverse 5'-3')- CCTGAGCTTCCTTCATCCCC
ATP2A2	(Forward 5'-3')- GAGCCTGAAATGGGCAAAGT<br>(Reverse 5'-3')- GGAACTTTGTCACCAACAGCAA
ADCY10	(Forward 5'-3')- GGCCATTGAGTTAGGCTCCC<br>(Reverse 5'-3')- TGTCGGTTGGGATTCTGGAG
PRNP	(Forward 5'-3')- ACCCTTTTGCGTGGTCCTTA<br>(Reverse 5'-3')- AAGTACATGCATATTTCAAAGACCT
RNP24/TMED2	(Forward 5'-3')- CATGGAAGTCCGGGAGAGAA<br>(Reverse 5'-3')- GGACCAAAGGACCACTCTGC
PEMT	(Forward 5'-3')- TTGCACGATGGGAACACAAG<br>(Reverse 5'-3')- AGAGTAGCAGGCCAGGTAGG

ARL6IP1	(Forward 5'-3')- AATAAGCCTGTCTTCCTATCTGGA<br>(Reverse 5'-3')- ACAGGATTAAAACTGCAAAAGTAGT
P4HA1	(Forward 5'-3')- TTCAAAGAGCTGGGGACAGG<br>(Reverse 5'-3')- AAAACAGTGGCTCCTCCTGC
RPN1	(Forward 5'-3')- GTAGCCTGCATCACAGAGCA<br>(Reverse 5'-3')- GGTCTCGTCAAAGTGACGGT
SYVN1/hrd1	(Forward 5'-3')- ACTGCCGCATTGTCTCTCTT<br>(Reverse 5'-3')- AGGATGCTGTGATAGGCGTG
ATP2C1	(Forward 5'-3')- CTTCTGGCGTGAGCTACGAG<br>(Reverse 5'-3')- ACTTGGTCTGGGATCTGGAAC
ELOVL3	(Forward 5'-3')- ATCCTCTGGTCCTTCTGCCT<br>(Reverse 5'-3')- AGCACAGTCCCCATAATGCC
AKAP6	(Forward 5'-3')- GTAGAATGTACCCCCTCCCCA<br>(Reverse 5'-3')- AGCCAGGCGTATCTTTCAGT
LMAN1	(Forward 5'-3')- TCAAAGAGCACCTGCACATAGT<br>(Reverse 5'-3')- GTTCTGGGCATTTCGGCTTT
lpcat3	(Forward 5'-3')- CTGCCGTCCTCACTACCTTT<br>(Reverse 5'-3')- TGCCGGTGGCAGTGTAATAG
AMFR	(Forward 5'-3')- ACTGCCCTGTGGACATCTTT<br>(Reverse 5'-3')- CTGCATGTTGGACAGGAGGT
EIF2AK3/PERK	(Forward 5'-3')- ACATCCTGCTTCTACAGCGT<br>(Reverse 5'-3')- CCACTTCTCATTGCCACTGC
SSR4	(Forward 5'-3')- CTATGCTGACGTCGGTGGAA<br>(Reverse 5'-3')- CACCTGATAACGCCCCACAT
HSD3B2	(Forward 5'-3')- CCAGTCCTTCCTCCAGGGAT<br>(Reverse 5'-3')- GACCCAGAAGAGGGCGTAAC
EXTL2	(Forward 5'-3')- ATGACCCTCCTGCTGTGAAG<br>(Reverse 5'-3')- TATGGCGGTGAACCCTTGAG
ERN1	(Forward 5'-3')- GATGCCTGCACCAATTCTGG<br>(Reverse 5'-3')- GCAGCCTGTATACGCTTGGA
SYPL2	(Forward 5'-3')- CCCTCTGCGATGAAGAGTCC<br>(Reverse 5'-3')- AGATGCCAAGGGTCACGAAG
CALU	(Forward 5'-3')- GTTGAAAACCAATGGCAGGAGT<br>(Reverse 5'-3')- GGATCATCCAGGTAAGTGCCAT
KDELR1	(Forward 5'-3')- TTCCGATTCCTGGGAGACCT<br>(Reverse 5'-3')- CGAGCGGGACTTCCAGATTT
EBP	(Forward 5'-3')- GACTGTCCCTGTGCTGGTTT<br>(Reverse 5'-3')- GTCTCCAAGCAGGTCTTCGT
CACNA1S	(Forward 5'-3')- CCATGCCGGAAGATGACAAC<br>(Reverse 5'-3')- GCGGCTTCAATCGAGAAGAC
RTN4	(Forward 5'-3')- AGAGGACAGATCACCATCTGCTA<br>(Reverse 5'-3')- ATAGGCTGGCACCAAACACC
SERP1	(Forward 5'-3')- TATGGCCAACGAGAAGCACA<br>(Reverse 5'-3')- GGGGCATTTCTCGAGGTCTT
DAD1	(Forward 5'-3')- GGGACCTTCCCCTTCAACTC<br>(Reverse 5'-3')- TCCGCTTTGTTCTGTGGGTT
CLN8	(Forward 5'-3')- CCTGGTCAGCAGCCTGTATC<br>(Reverse 5'-3')- TAGCGTAAGCAGAGCCAGTC
GAS1	(Forward 5'-3')- CACTCATGCAAGAAGTGGGC<br>(Reverse 5'-3')- AGGAGGGGAAAGGAGACGAG
DEGS	(Forward 5'-3')- CTGGCCATCTTTGCCCTTTG<br>(Reverse 5'-3')- AGGAACATGTAGTGCTCGGC
LMAN2	(Forward 5'-3')- ACTTCCAGGGCAGCACTATG<br>(Reverse 5'-3')- ATAGAGCCCTCTTTGCTGCG

FMO3	(Forward 5’-3’)- GAAGTGGCTCCTGGGTGATG<br>(Reverse 5’-3’)- AGTGACGAGCAGCATGTCC
UGGT2	(Forward 5’-3’)- AGAGTGCTGTAATTGCAAAGAACAT<br>(Reverse 5’-3’)- GCAATAATCCAGAGAGTGACTGC
RFT1	(Forward 5’-3’)- ACGCTGCTTTACTCAACCAC<br>(Reverse 5’-3’)- TGAGACATGCTCTGCGGAAG
SCAP	(Forward 5’-3’)- CCTCATCGGCTACTTCACCC<br>(Reverse 5’-3’)- CCCACGACAGCAAAGAGACA
ERO1LB	(Forward 5’-3’)- AGGGGCCAAGTCACTAAAGG<br>(Reverse 5’-3’)- AATCTGCATTTGTCACATCCAACA
ZMPSTE24	(Forward 5’-3’)- GCCAACCCACTCTTATTGGACT<br>(Reverse 5’-3’)- GCGGCTTAGGACTGTTAGGC
ACAT1	(Forward 5’-3’)- AGACGGGCTAACTGATGTCTAC<br>(Reverse 5’-3’)- AAGCGTCCTGTTCATTTCGT
ACSL4	(Forward 5’-3’)- CTCCGCTTACACTCTCTGACC<br>(Reverse 5’-3’)- TTTCCGGAACAGCAGCCATA
BCAP31	(Forward 5’-3’)- GTGTTGCTTCTCTGCATTCCC<br>(Reverse 5’-3’)- ACCAGCCGGGACTTGAAAAT
CES3	(Forward 5’-3’)- GATGTGCCCCCTGAGATGAT<br>(Reverse 5’-3’)- GCCTGGCATTTGGCTTGTG
EDEM1	(Forward 5’-3’)- CCCTGGACTGCAGGTGCTGA<br>(Reverse 5’-3’)- AGGGAGGGCACCATATCGTTT
P4HTM	(Forward 5’-3’)- CGCTGCAGGTTGTTCGATATG<br>(Reverse 5’-3’)- TACACAGGCCCACTGTCCA

Appendix Figure Legends

Appendix Figure S1: Molecular modeling data, ERMIT and AGR2 dimerization under basal and stress conditions. A) Root-mean-square deviation (RMSD) plot for the MD simulation of AGR2 WT and E60A mutant. The black and blue lines show the change in RSMD of CA atoms, whereas the green and yellow lines show the change in RMSD of CA atoms of the dimer domain (residue 54-70 of each monomer). The red line separates the equilibrium and production phases of the simulations. **B)** Radius of gyration plot of the dimer domain. AGR2 WT exhibits a stable radius of gyration, whereas the E60A mutant curves show an expansion after 100 ns, which implies dissociation of the dimer. Evolution of three distances between CA atoms of residues that form the edges (E59A-T67B and E59B-T67A) and middle (Y63A-Y63B) of the α-helices of monomer A and B of the dimer domain for **(C)** WT and **(D)** E60A mutant. The pattern is in agreement with the radius of gyration; for AGR2 WT, where the distances show small fluctuations to their initial values, which implies that the α-helices are held together. For AGR2 E60A mutant, the distances expand, which further demonstrate that the dimer dissociates. **E)** Last snapshot (200 ns) of AGR2 WT, which shows that the interactions between E60 and K64 of each monomer are retained. The α-helices of the dimer domain are highlighted with a pink arrow for monomer A and purple arrow for monomer B. **F)** Last snapshot (200 ns) of E60A mutant, which highlights the dissociation of the dimer. **G)** Western blot showing the expression of all the AGR2-IRE1cWT and AGR2-IRE1cKD constructs in HEK293T cells. **H)** Representative photographs of AGR2-IRE1cWT and AGR2-IRE1cKD localized to the ER compartment in HEK293T monitored by immunofluorescence. Calnexin was used as the ER marker. **I)** AGR2 dimer monitoring using ERMIT upon ER stress induced by increasing doses of DTT, thapsigargin or tunicamycin (the Y axis reports the ERMIT/XBP1 reporter alone ratio). **J)** IC50 values obtained for DTT, tunicamycin and thapsigargin on the dimerization of AGR2 using ERMIT.

Appendix Figure S2: **Functional analyses of the role of AGR2 dimer regulators. A)** Kinetics of AGR2 association with other possible binding partners as measured in Hela cells subjected to ^{35}S-methionine pulse-chase labelling. The top image

represents an X-ray exposure of the gel and the bottom image shows an immunoblot of AGR2 in the immunoprecipitate (AZC: azetidine 2-carboxylic acid). **B)** Graphical representation (heatmap) of the functional pathway analysis. The graph depicts the participation of most highly ranked genes (x axis) to clusters of semantic terms corresponding to the statistically significant, systemic functions (y axis). **C)** Graphical representation of the processes suggested as significantly enriched by functional pathway analysis, based on the Gene Ontology and Reactome annotations, of the 71 candidates. The proteins that are shown are also annotated as Endoplasmic Reticulum resident using Gene Ontology. AGR2 homodimer inhibitors are shown in red and enhancers in green. **D)** Representation of the AGR2 regulatory network as identified by us (Higa, Mulot et al., 2011) using proteomics. Indicated in color are the regulators of AGR2 dimerization (enhancers in green, inhibitors in red).

Appendix Figure S3: Co-IP and results from Protein-Protein Docking of AGR2 and TMED2 using FireDock. A) Co-immunoprecipitation of AGR2 with TMED2 under basal and tunicamycin induced ER stress in cultured HEK293T cells. **B)** AGR2 monomer (center) in color tan with N-terminal domain and dimer interface alpha-helix in red. Ten best ranked TMED2 configurations (different colors) clustering on left (2) and right (8) side of AGR2. None of the structures are positioned such that they can stabilize dimer formation. **C)** Electrostatic surface of AGR2 dimer with best docked TMED2 orientation (green). Arrows indicate interaction sites where complementarity to TMED2 electrostatic surface is identified. **D)** Upper panel: matching positive electrostatic area of TMED2 (brown arrow) to corresponding negative region in AGR2 dimer in (C); middle panel: electrostatic surface of TMED2 with complementary negative electrostatic surface at black arrow, matching the electrostatic positive patch of AGR2 dimer (black arrow in (C)); lower panel: electrostatic surface showing interaction of N-terminal region of TMED2, with corresponding N-terminal part of AGR2 (orange arrow in (C)). **E)** Close similarity in interaction between two docked TMED2 molecules (purple and green) with the AGR2 dimer. One TMED2 monomer interacting at each side of the symmetry-related regions of the dimer interface.

Appendix Figure S4: AGR2 in the ER proteostasis control. A) Immunoblot analysis of CD59-GFP WT and C94S expression in cells silenced for TMED2. The

loading was monitored with anti-p97 antibody and CD59 was detected with anti-GFP antibody. **B)** Immunoblot analysis of CD59-GFP WT and C94S expression in cells silenced for TMED2 and transiently overexpressing AGR2 WT or AA mutant protein. The loading was monitored with anti-actin antibody and CD59 was detected with anti-GFP antibody. **C)** Identification of the presence of AGR2 peptide binding sites on alpha 1 antitrypsin (A1AT). **D)** Secretion of A1AT in HuH7 cells under basal or tunicamycin-induced ER stress conditions in cells silenced or not for AGR2. **E)** Quantitation of intracellular and secreted A1AT in cells silenced or not for AGR2 using ^{35}S-methionine pulse-chase experiments and A1AT immunoprecipitation. **F)** Evaluation of MUC2 expression in HT29 cells upon modulation of AGR2 expression and ER proteostasis. The processing of a pool of MUC2 radiolabelled with 35S methionine was evaluated in cells silenced or not for AGR2**. G)** The impact of an AGR2 peptide-binding domain on the expression of MUC2 was evaluated in HT29 cells subjected to tunicamycin-induced ER stress.

Appendix Figure S5: **Association of AGR2 AA mutant with genetic modulation of TMED2. A)** Effect of ERAD-inhibiting drugs and chemical chaperon TUDCA on AGR2 intracellular expression as evaluated by Western blot. Actin (ACT) was used as loading control. **B)** Impact of TMED2 overexpression on intra- and extracellular AGR2 AA mutant expression (iAGR2 and eAGR2, respectively) in HEK293T cells as revealed by Western blot. Actin (ACT) was used as loading controls. **C)** Impact of TMED2 silencing (siTMED2) on intra- and extracellular AGR2 AA mutant expression (iAGR2 and eAGR2, respectively) in HEK293T cells as revealed by Western blot. Actin (ACT) was used as loading control. **D)** Expression and secretion of AGR2 WT by HEK293T cells upon treatment with brefeldin A (BFA, 5 µg/ml), bafilomycin A1 (baf, 200 nM), or both (B+B) for the indicated periods of time. Extracellular AGR2 (eAGR2), intracellular AGR2 (AGR2).

Appendix Figure S6: **Expression of AGR2 dimerization regulators in IBD samples. A)** Heat map representation of the expression of AGR2 dimerization regulators in a test IBD patient cohort. **B**) mRNA expression levels measured using quantitative reverse transcriptase-PCR analysis in colonic biopsies from healthy controls (CT), patients with colonic Crohn's Disease (CC) and patient with Ulcerative

Colitis (UC) from a validation cohort. P-values for Kruskal-Wallis non-parametric analysis are shown; Dunn's multiple comparison test vs. healthy controls. Note that RNP24 is an alternative name for TMED2.

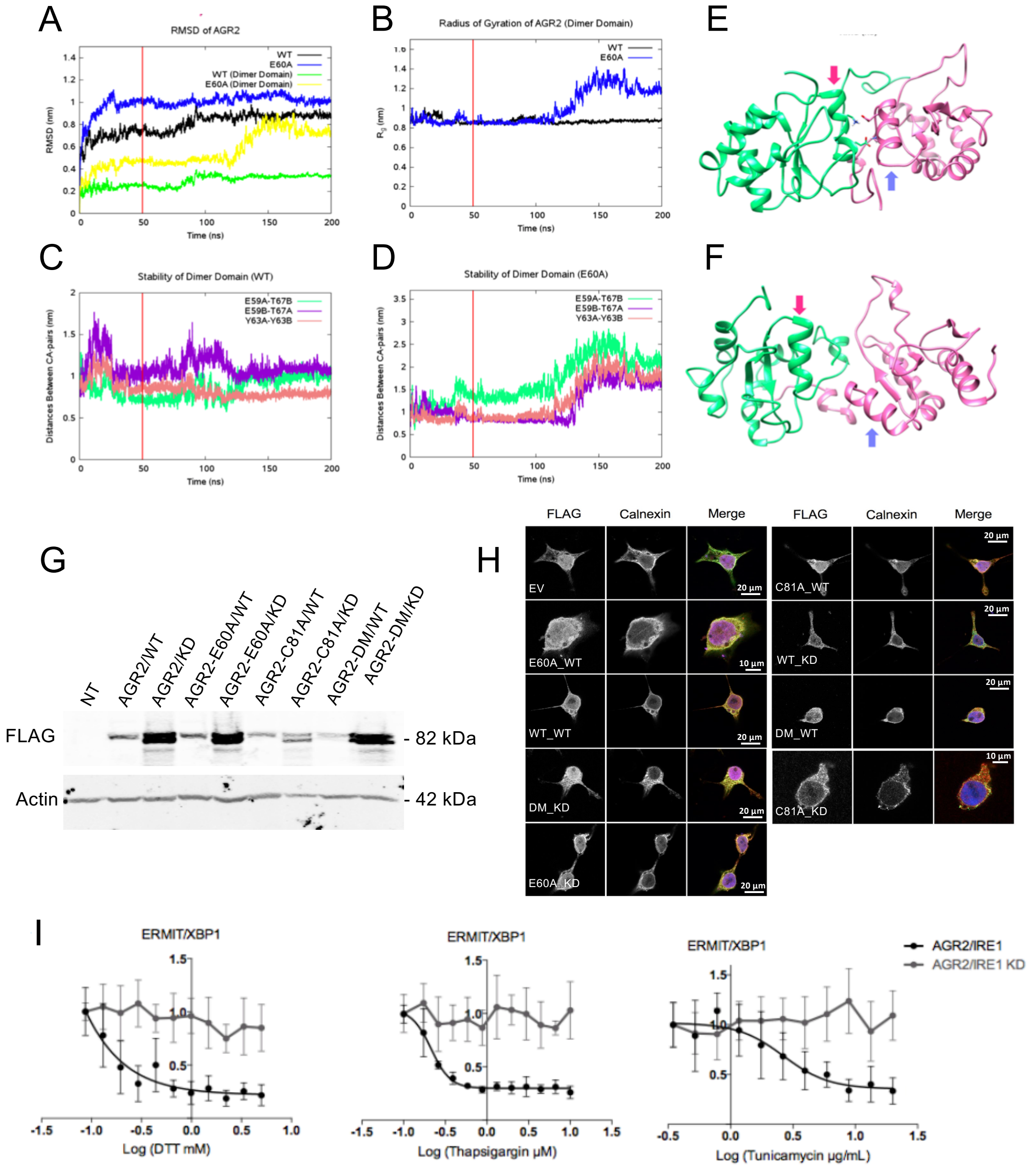

J

	DTT (mM)	Tg (µM)	Tun (µg/ml)
IC50	0.1 ±0.05	0.2 ±0.05	2.66 ±0.97

Appendix Figure S1

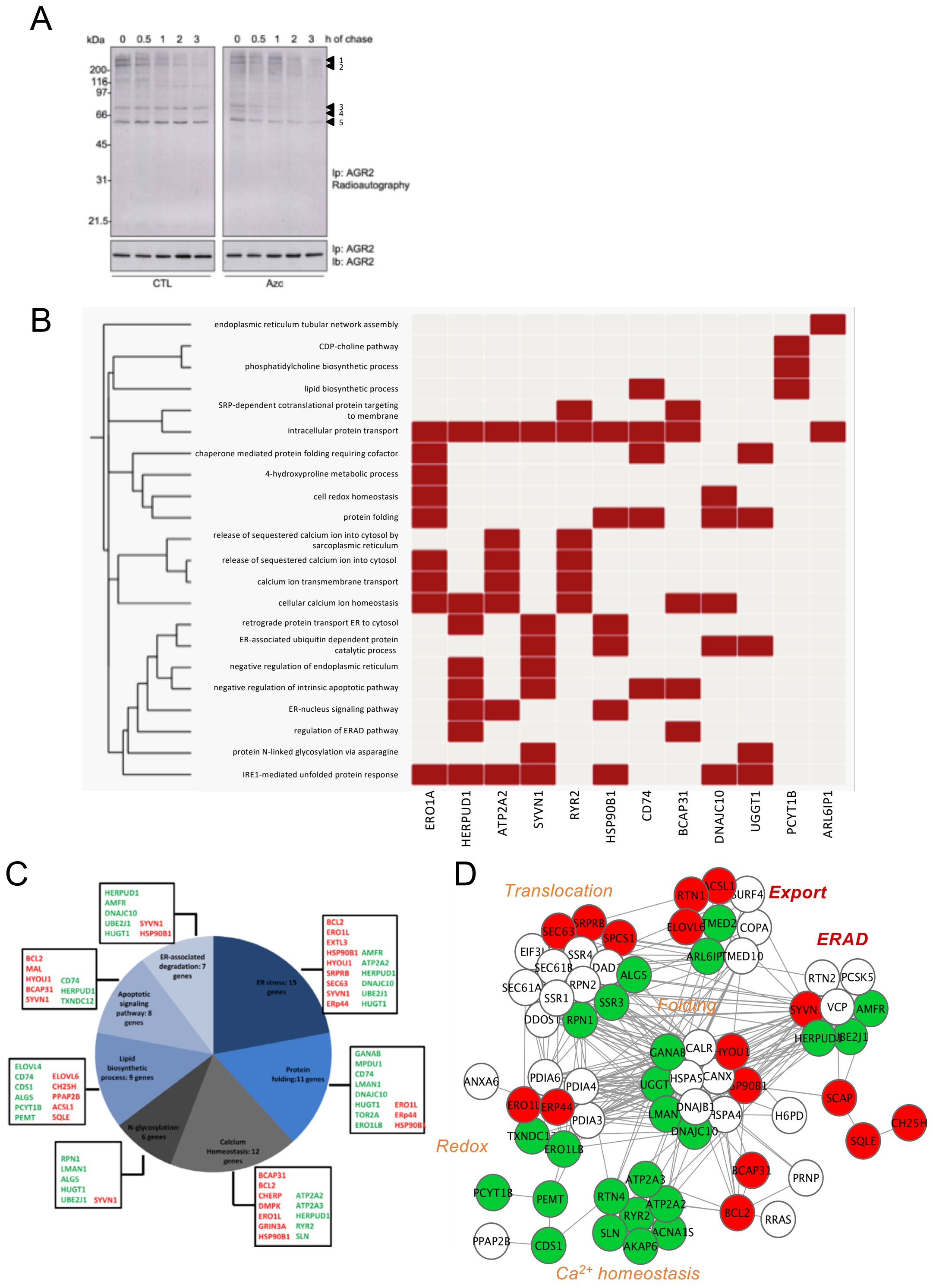

Appendix Figure S2

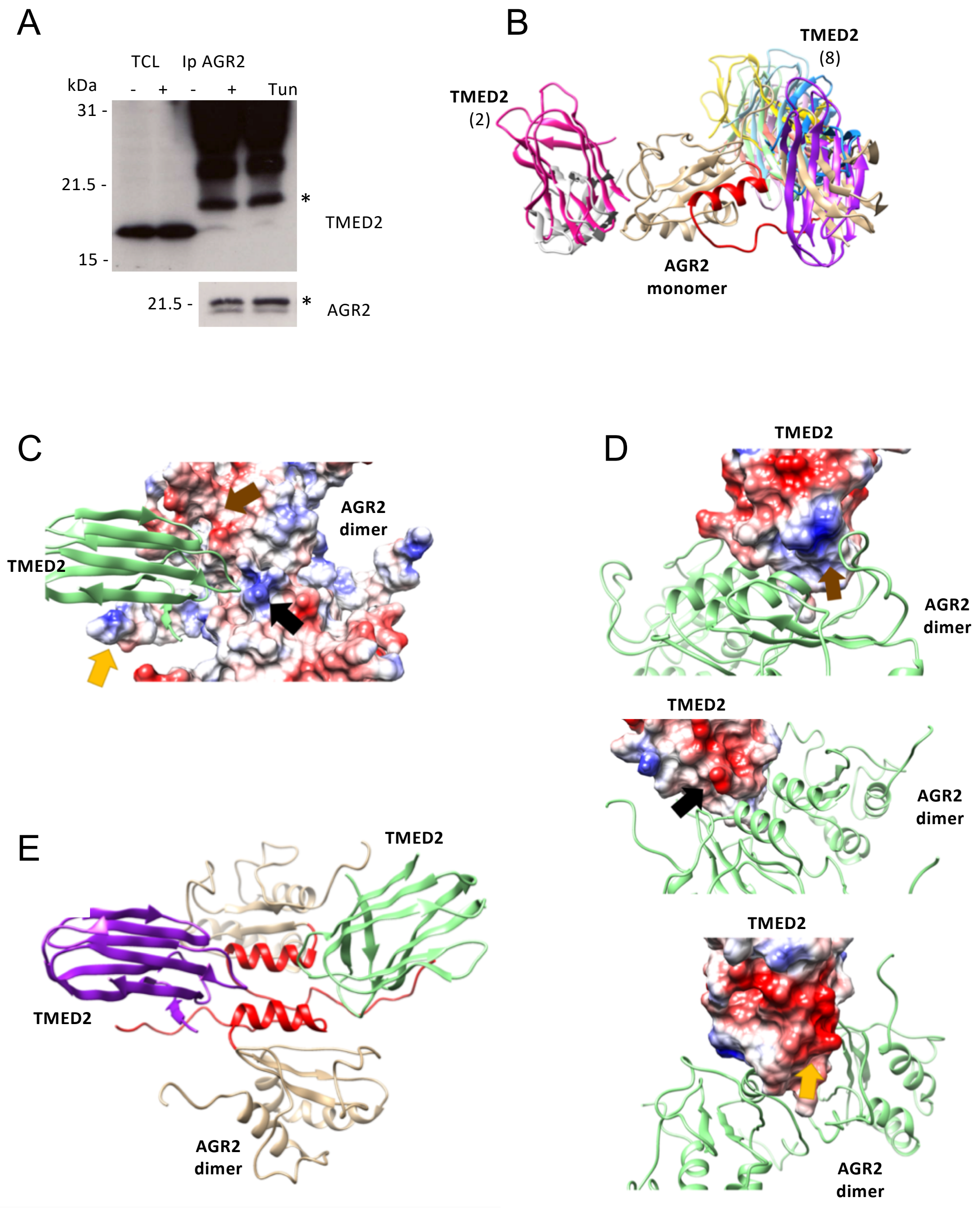

Appendix Figure S3

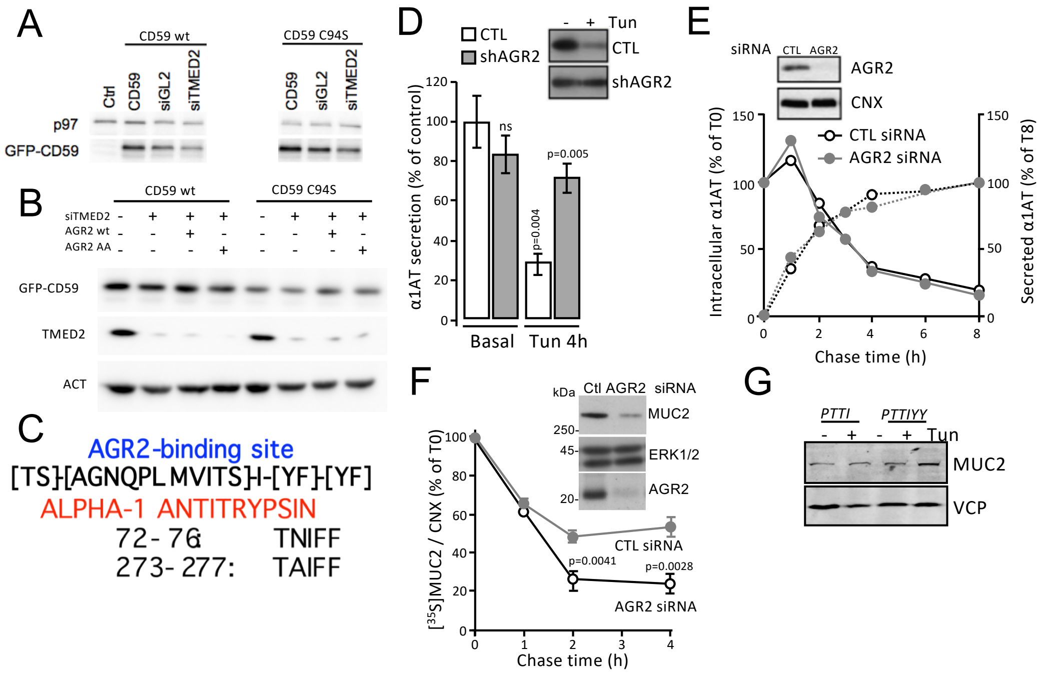

Appendix Figure S4

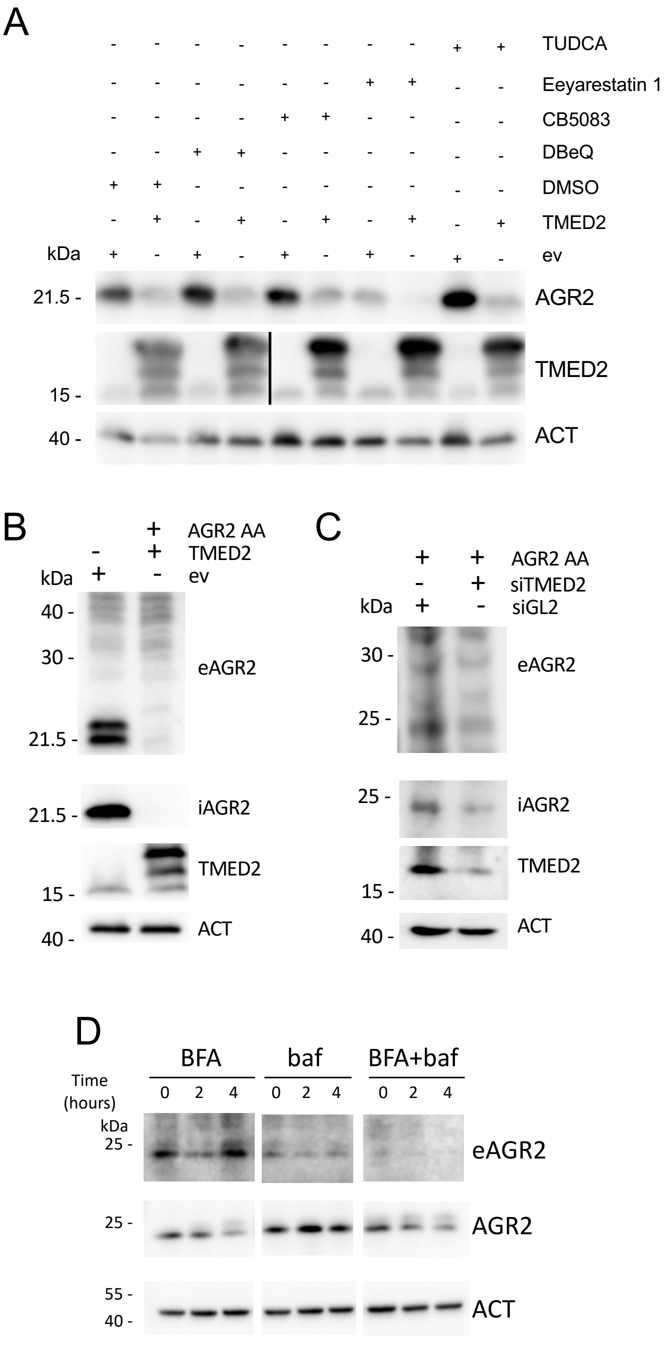

Appendix Figure S5

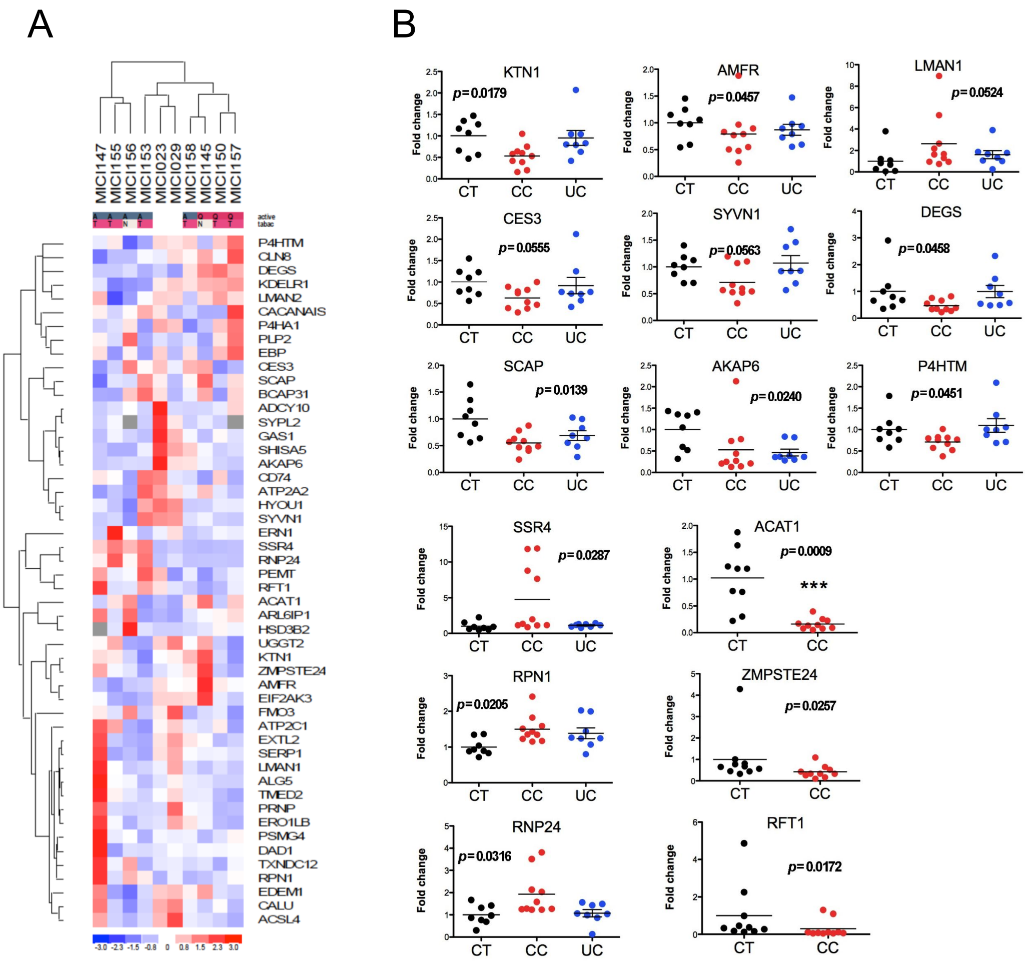

Appendix Figure S6

Appendix References

Higa A, Mulot A, Delom F, Bouchecareilh M, Nguyen DT, Boismenu D, Wise MJ, Chevet E (2011) Role of pro-oncogenic protein disulfide isomerase (PDI) family member anterior gradient 2 (AGR2) in the control of endoplasmic reticulum homeostasis. *J Biol Chem* 286: 44855-68
